# Udder Health Monitoring for Prevention of Bovine Mastitis and Improvement of Milk Quality

**DOI:** 10.3390/bioengineering9110608

**Published:** 2022-10-23

**Authors:** Andra-Sabina Neculai-Valeanu, Adina-Mirela Ariton

**Affiliations:** Research and Development Station for Cattle Breeding Dancu, Laboratory of Nutrition, Quality and Safety of Food Products, 700490 Iasi, Romania

**Keywords:** dairy cattle, mastitis, heat stress, animal welfare, animal health, non-invasive

## Abstract

To maximize milk production, efficiency, and profits, modern dairy cows are genetically selected and bred to produce more and more milk and are fed copious quantities of high-energy feed to support ever-increasing milk volumes. As demands for increased milk yield and milking efficiency continue to rise to provide for the growing world population, more significant stress is placed on the dairy cow’s productive capacity. In this climate, which is becoming increasingly hotter, millions of people depend on the capacity of cattle to respond to new environments and to cope with temperature shocks as well as additional stress factors such as solar radiation, animal crowding, insect pests, and poor ventilation, which are often associated with an increased risk of mastitis, resulting in lower milk quality and reduced production. This article reviews the impact of heat stress on milk production and quality and emphasizes the importance of udder health monitoring, with a focus on the use of emergent methods for monitoring udder health, such as infrared thermography, biosensors, and lab-on-chip devices, which may promote animal health and welfare, as well as the quality and safety of dairy products, without hindering the technological flow, while providing significant benefits to farmers, manufacturers, and consumers.

## 1. Introduction

Over the past decades, dairy farmers have improved the genetics of their cattle by focusing on sustainable farming practices to produce more milk with fewer cows and lower the amount of greenhouse gas (GHG) generated per liter of milk produced [1,2]. Although they resemble their predecessors, due to the improved genetics, the increase in milk yield is almost double compared to 50 years ago [3,4,5]. Breeding cattle for higher milk production is associated with a higher incidence of mastitis and increased somatic cell count, specifically, leukocytes, secretory cells, and squamous cells transported into milk as a reaction to intramammary infection (IMI) [6].

Mastitis is the most frequently occurring infectious disease in dairy cattle, with worldwide economic losses estimated to be more than USD 40 billion USD per year [7]. Udder health is of paramount importance for sustainable milk production. Mastitis is more than just a medical condition; it impacts milk quality, cattle performance, and farm antimicrobial use [8].

The diagnosis of subclinical mastitis in dairy cows, as well as the early detection of clinically expressed mastitis that induces macroscopic changes in milk secretion, is an essential component of any program established to improve the health parameters of a dairy farm. This goal is of tremendous economic interest, both for farmers and manufacturers, given that a large number of somatic cells in milk and the presence of bacteria have a severe negative influence on milk production and quality [9,10].

The EU’s largest funding mechanism, the new Common Agricultural Policy (CAP), is focused on ensuring farmers’ economic viability, resilience, and incomes. Additionally, it aims at improving environmental conditions and the development of rural areas. Since the link between animal health and welfare and food safety is well documented, one of the main objectives of the new CAP is to improve the response of EU agriculture to society’s food and health requirements. This includes providing safe, nutritious animal products produced sustainably, with higher animal welfare standards [11,12,13]. Thus, using non-invasive tools for monitoring udder health and prevention of bovine mastitis may enable the transition to a “low-antibiotic farm model” with healthier animals and safer dairy products.

This article reviews the impact of heat stress on milk production and quality. It emphasizes the importance of udder health monitoring, with a focus on the use of emergent methods for monitoring udder health, such as infrared thermography, biosensors, and lab-on-chip devices, which may promote animal health and welfare, as well as the quality and safety of dairy products, without hindering the technological flow, while providing significant benefits to farmers, manufacturers, and consumers.

## 2. Impact of Heat Stress on Udder Health, Livestock Production, and Milk Quality

### 2.1. Dairy Industry in the Era of Climate Change

**Figure 1:**
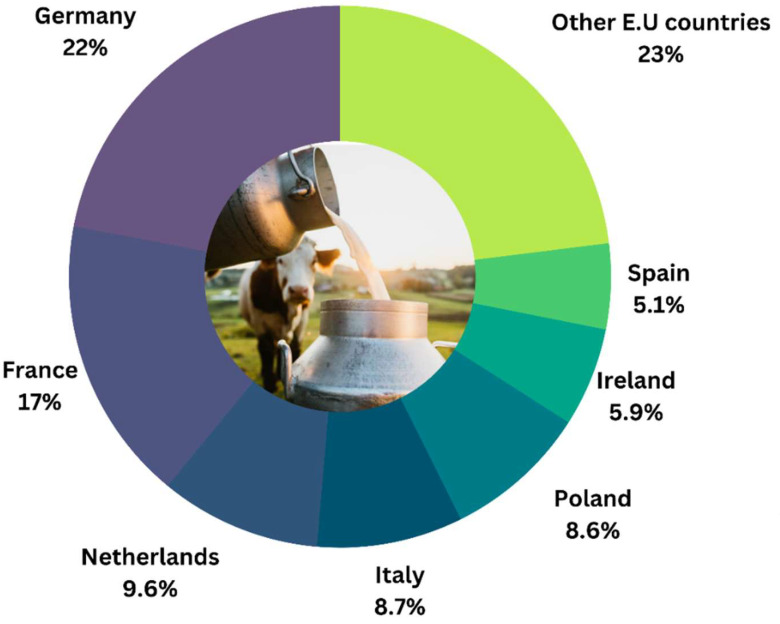
The graph shows the percentage of milk produced by EU countries (except Luxembourg) in 2020 (Eurostat, 2021).

The dairy industry is a driving factor in the European Union’s agricultural economy (EU), with 145 million tonnes of cow’s milk produced in 2020 [14]. Even though milk is produced in all EU member states, milk production from seven nations accounted for about 77 percent (119 million tons per year) of the total raw milk produced in the EU [14] (Figure 1).

Notwithstanding the considerable progress in approaches to reducing heat stress’s impact on animal production, heat stress poses a significant barrier to global livestock production sustainability, inflicting significant economic losses to livestock sectors in the warmer regions of the world [15]. The physical environmental alteration, dietary interventions, and genetic selection for stress tolerance are all potential options for mitigating some detrimental effects of heat stress. While genetic selection for heat tolerance would be the long-term method for producing heat-robust future animals, identifying heat-tolerant animals is challenging due to a negative relationship between production characteristics and heat tolerance. Dairy cattle have been bred and genetically modified to make more milk, and they are fed massive amounts of high-energy feed to sustain their increased milk output. This has increased the dairy industry’s efficiency, income, and milk production [16,17].

The impact of high temperatures on animal welfare is a growing concern, especially in light of projected global warming scenarios. The Earth’s temperature is anticipated to rise by 1.5 °C over the next few decades (IPCC 2018), and extended episodes of extremely hot weather are expected to grow in frequency, severity, and length [18]. In this climate, which is becoming increasingly hotter, millions of people depend on the capacity of cattle to respond to new environments and to cope with temperature shocks, as well as additional stress factors such as solar radiation, animal crowding, insect pests, and poor ventilation [19].

Given the unfavorable association between production characteristics and heat tolerance, the ongoing selection for enhanced productivity has resulted in a steady decline in heat tolerance throughout the last 50 years. Moreover, in the twenty-first century, milk production loss owing to heat stress is predicted to grow at a pace of over 170 kg/cow/decade [16]. The productive capacity of the dairy cow is under increasing pressure as the demand for milk rises to meet the needs of a rising global population.

### 2.2. Assessing Heat Stress in Cattle

Heat stress is a confluence of environmental variables such as temperature, relative humidity, solar radiation, air movement, and precipitation that affect the welfare and productivity of dairy and beef cattle [19]. This condition arises in dairy cattle when there is a difference between the heat energy generated by the animal and the heat energy lost to the environment.

**Figure 2:**
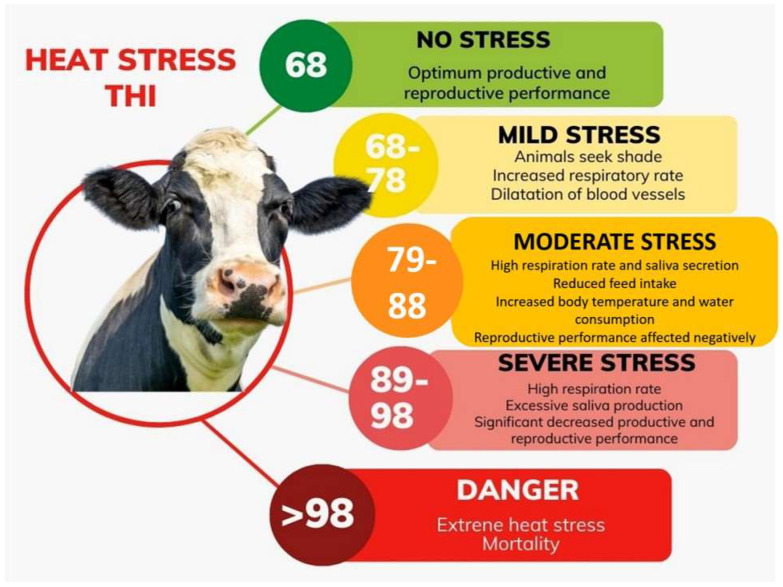
The severity of heat stress in dairy cattle in relation to the THI index.

Several indices have been developed to assess heat stress by incorporating various environmental parameters. The temperature-humidity index (THI), which combines the impacts of air temperature and humidity associated with the amount of thermal stress, is the most extensively used environmental indicator for monitoring and reducing heat-stress-related losses [20,21]. Several models of equations have been developed to assess THI. The equation developed by the National Research Center in 1971 [20,22],

*THI* = (1.8 × *AT_avg_* + 32) − [(0.55 − 0.0055 × *RH_avg_*) × (1.8 × *AT_avg_* − 26)

where *T* = dry bulb temperature (°C) and *RH_avg_* is daily mean relative humidity (%) is regarded as the most appropriate and is routinely used in heat stress studies conducted in several climates [23,24]. The strong relationship between THI and a variety of variables, including heart rate, respiration rate, rectal and vaginal temperature, and dry matter intake (DMI), has been highlighted in the literature [25,26,27] (Figure 2).

Thermal balance is considered to be influenced by a series of factors such as genotype, diet type and structure, body condition, fat distribution and deposition, development and lactation, health status, and degree of adaptation [28,29,30].

The relationship between THI and milk yield may be assessed using a reference equation provided by Berry et al. (1964) [31]. According to the authors the:

Decline in milk yield (kg/d) = − 1.075 – 1.736 × *NL* + 0.02474 × *NL* × *THI*

where *NL* is the usual daily milk production (kilograms per day) measured between the optimal temperature level for cattle, considered to range between −13 °C −/+ 25 °C [27,29,30] and *THI* is the daily mean temperature–humidity index. Using this equation, it is observable that the daily milk yield (kilograms per day) decreases as the temperature–humidity index increases, especially for higher-productive cows. According to a study conducted by Bernabucci et al. (2010) [32] a loss of approximately 0.27 kg of milk may be observed for each successive unit increase in the temperature–humidity index. Moreover, the impact of heat stress extends beyond milk production, influencing its quality simultaneously as well, with considerable modifications being reported in the case of certain physical-chemical parameters, such as lipid, lactose, protein, casein, and urea content [27,33,34,35]. According to studies carried out by Hill and Wall (2014) [36], Liu et al. (2017) [37], as well as Dado-Senn et al. (2021) [38], a reduction in lactose, casein and/or fat content was observed in cows exposed to heat stress.

Furthermore, pathogen multiplication is most definitely favored by changes in environmental conditions, thus increasing the microbial burden in raw milk. The elevation in average temperatures caused by climate change is forecast to increase the prevalence of bovine mastitis in dairy farms, leading to greater economic losses [39].

During the warmer months, there is a likelihood of an increase in the number of cases of mastitis in a herd [40,41,42]. Thus, the immunological competence of lactating and dry cows might decrease when they are subjected to heat stress. This may have a detrimental impact on udder health [43]. A cow’s immune system might be suppressed for an extended length of time throughout the productive years of its life, which may negatively affect how cows react to being exposed to pathogens [44,45].

The inflammatory process triggered by a mammary infection alters the permeability of the blood-mammary gland barrier, allowing more ions, proteins, and somatic cells to enter the milk. Prior studies have shown that the number of somatic cells substantially affects milk production, in addition to the amount of protein and lactose in milk [46,47,48,49,50,51,52]. The decrease in milk production that occurs concomitant with a rise in milk SCC is primarily the result of physical damage to the epithelial cells responsible for producing milk [53].

The damage to the alveolar epithelial cells has also been suggested as a possible cause for the lactose decrease. Since lactose contributes significantly to ensuring the osmotic pressure of milk, a decline in its amount triggers a significant loss in milk production. Furthermore, sodium and chloride ions are transferred from the blood into milk to preserve the osmotic equilibrium, raising their overall content to an abnormally high level [54,55]. In addition, an increase in proteins may be attributed to a disturbance in the integrity of the mammary epithelium caused by bacterial toxins.

### 2.3. Discussions

The dairy cow’s immune system is a crucial factor in determining the occurrence of clinical mastitis and the severity of the symptoms. While various management and environmental conditions may impair the immunological system and production in dairy cattle, heat stress has the most detrimental impact on animal health and production. Consecutive to heat stress exposure, the proliferation of the epithelial cells from the mammary gland is inhibited. Thereby, heifers born to cows exposed to heat stress in late gestation have a lower milk production potential. Heat stress also dramatically modifies the immunological response of calves and cows from the fetal stage through lactation. Heat stress also dramatically modifies the immunological reaction of calves and cows from the fetal stage through lactation.

When exposed to heat stress, a cow’s body undergoes metabolic and hormonal changes that depress the immune system and have a detrimental effect on the amount and function of polymorphonuclear neutrophil granulocytes (PMN). Immunoglobulin G, the quantity of PMN, and their function are all components of the innate immune system, which is the initial line of defense against pathogenic organisms. The mammary gland becomes more susceptible to infections by altering the number of PMNs and their activity. As a reaction to the bacteria’s growth, the number of somatic cells, primarily white blood cells mobilized to fight the infection, increases.

The likelihood of mastitis is influenced by the cow’s exposure to pathogens, the local and systemic defense mechanisms, and environmental factors. The clinical manifestations of severe mastitis might differ from cow to cow, depending on the kind of bacteria present, the number of microorganisms, as well as the immunological response of the cows. Symptom intensity is primarily determined by the interplay between predisposing factors, innate immunity, the functional capability of glandular tissue in the udder, and the success of mastitis treatment.

## 3. Udder Health Management—A Key Role in Ensuring Milk Quality

Dairy farming has progressed over several decades and is now a vital source of high-quality foods and revenue for many of the world’s population. Nonetheless, it must continue to develop to ensure the sustainable production of dairy products that fulfills the demands of a growing global population [56]. Every dairy farm strives to value the production potential of its animals and produce as much high-quality milk as possible. Poor milk quality impacts the dairy business, resulting in decreased manufacturing potential and shorter shelf life for milk and dairy products [57,58,59].

Milk quality is a concept that encompasses the nutritional, physical-chemical, hygienic-sanitary, and organoleptic properties. Milk somatic cell count (SCC) is used as a marker in all developed countries to assess the incidence of mastitis in dairy herds, inform processors regarding the quality of raw milk, and quantify farm-level hygiene. Among the several milk quality screening assays available, the estimation of milk SCC is the most reliable test for detecting the asymptomatic type of mastitis (subclinical mastitis). In some countries, producers receive reimbursement for delivering milk with a low somatic count due to its more desirable technical characteristics and a longer shelf life [54].

A dairy cow’s lactation may be classified into three distinct stages: early, middle, and late. Milk is continually generated by milk-secreting epithelial cells in a lactating mammary gland. However, the highest milk production yield is observed in the early lactation stage. Subsequently, a physiological reduction in milk production may be observed as the lactation continues. Previous research has shown that the SCC is psychologically more significant in the first few weeks after calving and rapidly declines between 25 and 45 days after that. Afterward, it may steadily increase again during the late lactation stage [60,61].

Ensuring the quality of milk and dairy products remains challenging, especially if effective means and strategies for monitoring udder health and preventing bovine mastitis have not been appropriately implemented [62,63].

Bovine mastitis, or udder inflammation, is the costliest disease determining significant losses across European dairy farms. It affects almost half of all cows at some point in their lives, even in those farms with proper hygienic procedures. Yet, its prevalence varies widely from one farm to another [64,65]. Udder inflammation is frequently regarded as the greatest challenge to the dairy industry, resulting in financial losses and negative public health implications. During lactation, illness in one-quarter of the udder may reduce milk yield by at least 10%. Additionally, mastitis is also the leading cause of premature culling in dairy cows. Thus, financial losses associated with mastitis are linked to the expenditure of drugs, veterinarian services, laboratory expenses, and additional labor for farmers.

Losses are also incurred due to diminished milk production, discharge of milk due to antibiotic residues, and reduced efficiency in the manufacturing of dairy products [9,66,67]. Processing milk with non-compliant hygiene standards causes challenges in producing different dairy products, a commensurate decrease in cheese yield, a deterioration in taste, and implicitly, a decline in market competitiveness for dairy manufacturers [68,69,70].

Apart from farmers and dairy products manufacturers, consumers are negatively affected by poor quality or a lower supply of dairy products, meaning they would not benefit from their high nutritional quality, palatability, and safety, which may promote good health and well-being. Previous studies have highlighted the strong relationship between the food chain safety and security, respectively, and the welfare and health of animals [71,72,73,74,75]. Stress and poor welfare might enhance an animal’s vulnerability to pathogenic organisms; thus consumers may be at risk of contracting common food-borne diseases such as *Salmonella*, *Campylobacter*, and *E. coli*. The welfare and health of animals reared for food production are greatly influenced by farm management [76,77,78].

As in the case of mastitis, an increase in the number of somatic cells and bacteria in raw milk also signifies an increase in the activity of proteolytic and lipolytic enzymes in milk. Plasmin, for example, is a caseinolytic enzyme synthesized from plasminogen, which develops in the blood and most likely enters the milk due to the destruction of the mammary epithelium. Casein degradation will generate foul-smelling metabolites that will replace the pleasant fragrance associated with fresh milk [79,80] and poor curding, reducing the amount of cheese that may be manufactured [54,81]. A study by Charismiadou et al. (2015) [82] showed that the plasminogen activator activity might be four times higher per cell in animals with high somatic cell counts. Through the activation of their endogenous enzymes, somatic cells have been demonstrated in several recent studies to affect cheese’s technical characteristics and overall quality [79,81,83]. Since these enzymes are not completely inactivated by pasteurization, the proteolytic and lipolytic processes may continue even after pasteurization and preservation under refrigeration conditions; thus, the shelf life of milk and derived dairy products will be considerably reduced [84].

In light of these food safety concerns, udder health management is critical for dairy production systems, the efficient control of mammary inflammations being an essential tool in minimizing foodborne disease and providing nutritious dairy food products [85,86,87] Several factors play a crucial role in the occurrence of bovine mastitis in farms, including microorganisms, immune responsiveness, environment, barns, milking parlors, cleanliness, nutrition, and of course, humans [6,88,89,90,91].

Antibiotics have conventionally been seen as the primary line of attack against bacterial infections in dairy cattle, particularly in the event of mastitis, whereby antibiotic residues may be found in the milk, and there is a risk of microbial resistance spreading to the environment. Since the spread of multiple antibiotic-resistant (MAR) bacteria represents a significant public health problem for animal and human health, as well as food security, there is currently a key focus on reducing the consumption of antibiotics in livestock farms for transition to a “low-antibiotic farm model” [3,65,67].

Setting SMART (Specific, Measurable, Acceptable, Realistic, and Time-bound) goals is the first step in the process, followed by developing an action plan to achieve these objectives [56]. Production of bovine milk with hygiene parameters that meet EU regulations is a realistic and achievable goal, given that veterinarians, specialists, and farmers have the requisite expertise and collaborate properly [11,92].

### Discussions

When creating farm goals, it is imperative to adopt a step-by-step approach and accordingly plan to guarantee that each objective is completed appropriately and that these strategies are integrated into standard operating procedures. Therefore, the early detection of bovine mastitis is crucial for fast and effective therapy of the disease.

The assessment of management practices on a routine basis, as well as the collection and analysis of data in real-time, are all components of udder health monitoring. The primary goal in udder health management is to govern essential control points, such as cleanliness, body condition, teat end condition, milk parameters, and medical interventions, in such a manner that the outcomes (udder health parameters) are always at their best. However, since mastitis is a disease caused by several factors, it is impossible to completely avoid all challenges associated with it in real life. As a result, data regarding the health of the udder are also consistently examined to identify irregularities before they evolve into complications of clinical nature.

When udder health data and management are quantified, a farm is run more similarly to a business. This involves paying close attention to maximizing productivity, carefully considering the procedures involved, establishing transparent commitments and objectives, and conducting thorough evaluations of the procedures and the outcomes.

## 4. On-Farm and Lab Methods for Monitoring Udder Health and Milk Quality

**Figure 3:**
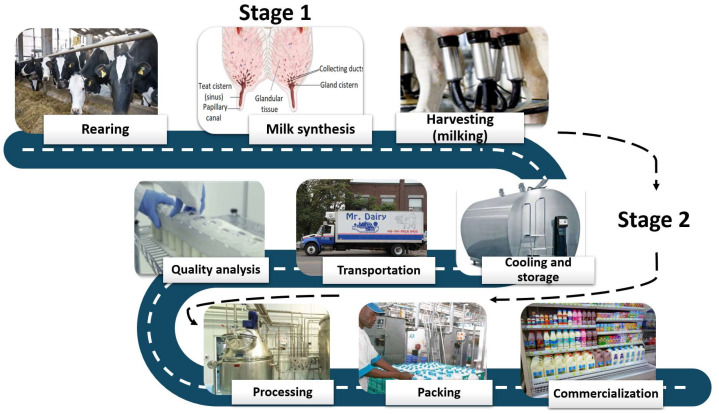
Milk way—from the rearing to the consumer.

From its origin to the point of consumption, milk moves through two separate stages [93]. The first stage extends from the mammary alveolar tissue to the papillary orifice of the galactophore (papillary) canal. In contrast, the second stage covers the way from the milking machine to the consumer (Figure 3).

The essential thing to mention in this respect is that the current set of mandatory tests for milk quality control pertains only to the second stage of the milk journey; thus, only the existing hygienic-sanitary circumstances in this phase after milking are shown. Thereby, the two main parameters, somatic cell count (SCC), respectively total bacteria count (TBC), may offer different types of information regarding udder health and milk quality [94]. In general, the presence of inflammation and subclinical mastitis is indicated by SCC levels of more than 200,000 cells/mL of milk. In most European nations, the limit for farm milk commercialization was set at 400,000 cells/mL, while in the United States, the limit is set at 750,000 cells/mL, and the price of milk decreases as the amount of SCC cells gets closer to the legal limit [54,92,95].

According to Regulation (EC) No 853:2004 of the European Parliament and the Council, raw milk must originate from animals that do not exhibit any evidence of contagious diseases that may be transmitted to people, as well as any indicators of illnesses of the mammary or genital tract that might potentially contaminate milk. The total bacteria count and the bulk milk somatic cells are the two primary health regulatory standards taken into consideration for assessing milk quality. Somatic cell count is defined just for raw cow milk, and it corresponds to a cell count of fewer than 400,000 cells per milliliter. In contrast, the plate count at 30 degrees Celsius must be less than 100,000 forming colony-forming units per milliliter. These two regulatory standards are each stated as a rolling geometric average over two or three months, with at least one sample collected per month [96]. The requirements for testing total bacteria and somatic cell count are outlined in Regulation (EC) No. 2074/2005, revised by Regulation (EC) No. 1664/2006 regarding the implementation of specific measures for original animal products intended for human consumption. The requirements for testing total bacteria and somatic cell count are outlined in Regulation (EC) No. 2074/2005, revised by Regulation (EC) No. 1664/2006 regarding the implementation of specific measures for original animal products intended for human consumption. The reference methods are EN/ISO 4833 for the plate count at 30 degrees Celsius, respectively ISO 13366-1 for the somatic cell count [97].

Increased SCC always indicates the presence of infection in some mammary quarters, whereas TBC mostly indicates milking hygiene or sanitation problems [98,99,100]. The presence of elevated SCC in the second phase of the milk route suggests the existence of an undesirable scenario in the herd, most likely owing to bovine mastitis. Increased SCC has a negative impact on the organoleptic characteristics of milk and its appropriateness for producing quality products [101,102,103,104], whereas increased TBC in raw milk represents, among other things, the risk of food-borne disease for consumers [105,106,107,108]. If both limits are surpassed, the milk is entirely inadequate in terms of quality, both nutritionally and hygienically, economically, and technologically. Screening the herd somatic cells count levels weekly, as an integrating part of the milk quality monitoring procedure, may provide farmers with useful information regarding the potential going ongoing herd-level disease, as well as the effectiveness of the implemented barn and milking hygiene procedures.

### 4.1. Conventional Methods for Monitoring Udder Health

The effectiveness of the milking routine and the performance of the milk collection equipment has a crucial impact on milk quality and udder health in dairy cattle [109]. The teat canal acts as the main physical barrier that prevents bacteria from entering the udder via the teat canal. Between milkings, the smooth muscles surrounding the teat canal should be constricted, and the teat canal should be securely closed to prevent infections from entering the teat canal and, from there, the udder [110]. This defense mechanism is reinforced by the presence of keratin cells, rich in lipids, present inside the teat canal. When the skin is elastic and smooth, without any lesions, the teat is in the best position to provide a natural barrier against the invasion of pathogens that cause mastitis. This is because the teat’s skin is more likely to withstand the pressure of the pathogens.

Any stress applied to the teats, even for a very short period, might affect their inherent capability to withstand a pathogen invasion. While the majority of attention is focused on teat-end hyperkeratosis, other short-term teat disorders, such as discoloring, sores, edema, and congestion, indicate poor milking performance [111,112].

Teat scoring, more accurately known as teat-end scoring, is a helpful technique to assess the amount of teat-end hyperkeratosis and other teat lesions in a dairy herd (Table 1). This method may be a useful management tool for farmers, providing information regarding the efficiency of the milking equipment and the milking procedure [113]. Research has shown that a circulatory impairment of any kind may be connected with an increased risk of a mastitis infection that is not yet clinically apparent [113]. Mastitis has been linked to teat hyperkeratosis, which is thought to cause alterations in the surrounding tissue of the teat canal, enabling bacteria to enter easily into the mammary gland. For this reason, it is essential that the assessment of teat-end scoring be carried out at regular intervals on at least 20% of the herd so that changes may be monitored over time [114] before the quality of milk is hindered [109].

Several tests have been developed to identify some of the changes that take place in milk yield throughout the progression of a mammary gland infection [115]. The majority of the tests pursued to reveal certain physicochemical modifications, such as an increase in the number of somatic cells (SCC) by direct or indirect counting (tests based on organic detergents: California Mastitis Test and similar tests such as the Wisconsin Mastitis Test, R-mastitest), the accumulation of chlorides, an increase in pH, electroconductivity (EC), viscosity, or catalase, an increase in udder skin surface temperature, the presence of grains with a diameter 0.1 mm.

Mastitis causes alterations in the real ionic dynamics of vascular components due to excessive cellular destruction and weakened milk–blood barrier. Loss of intracellular potassium results in a rise in the amounts of sodium, potassium, calcium, magnesium, and chloride ions in the blood, while the concentration of potassium ions declines. The electro-conductivity (EC) of milk is altered, and the pH is elevated due to these processes. These variations serve as a diagnostic sign for distinguishing milk with unusual qualities. Due to its simplicity and rapidity, with a cost/sample almost equal to the cost of the equipment, the electrical conductivity (EC) of milk has been studied extensively for the detection of clinical mastitis in the past [116]. The research conducted by Khatun et al. (2022) [117] highlighted that mastitis detection systems that rely only on EC are unlikely to accomplish the appropriate sensitivity and specificity criteria, but improvements are possible if several measures are performed (Table 1).

On the other hand, a study carried out by Kandeel et al. (2019) [55] showed that milk sodium, potassium, and calcium concentrations, as well as EC, were not sufficiently accurate to diagnose subclinical mastitis (SCM) and intramammary infections (IMI) in cattle, therefore they cannot serve as routinely udder health monitoring tools. Milk pH testing has also been proposed as a simple, inexpensive, and useful on-farm approach for identifying SCM and IMI in cattle. However, different authors concluded that milk pH does not offer a clinically effective technique for identifying SCM or IMI in dairy cattle [118,119].

Multiple kinds of predictive variables were proposed by Kamphuis et al. (2008) [120] as a means to enhance mastitis detection performance. Modifications in milk yield, milk temperature, milk color, cow activity, and other milk components are further markers used in the diagnosis of mastitis [119,121]. Using numerous criteria allows for a more accurate prediction of mastitis status, as shown in research by Khatun et al. (2018) [122]. It is also expected that their usefulness in farms will increase if more precise detection technologies are developed that use various measurements.

Currently, the California Mastitis test (CMT) and similar tests are routinely used by small and large-size farms to assess udder health. However, due to the subjectivity of reading and interpreting the results, they give less reliable results than the direct counting of somatic cells but more correct than other methods. The advantage of this method is that it is relatively fast, less expensive, and within reach of any farmer [123]. The favorable reviews enjoyed by CMT and similar tests are probably also because they were the first in the category of those simultaneously assessing two changes from two different categories: the number of cells and the pH [10,124] (Table 1).

Infections of the udder may also be identified by examining many additional biomarkers, such as secreted enzymes that indicate tissue damage. Colorimetric and fluorometric assays may be used to determine the activity of lysosomal N-acetyl-β-d-glucosaminidase (NAGase) or lactate dehydrogenase (LDH) in milk. A significant fraction of the enzyme NAGase is generated by epithelial cells of the udder that have been injured; such is the case of mastitis. According to a study conducted by Hovinen et al. (2016) [125], NAGase activity may be a reliable indication of both subclinical and clinical mastitis. Due to the breakdown of the blood–milk barrier that occurs after an intramammary infection, there is a rise in the amount of immunoglobulin G (IgG) found in the milk. Both lactate dehydrogenase (LDH) and serum albumin (SA) can cross the aforementioned barrier; hence, both may be utilized as indicators to predict the IgG transfer into milk and, subsequently, the presence of an intramammary infection [117]. One of the commercially available methods for assessing the LDH activity is the UdderCheck^TM^ from PortaCheck, which utilizes paper-based test strips and evaluates the color changes in the presence of an LDH-specific substrate. The severity of the disease is determined by making a qualitative comparison of the results using a color chart (www.portacheck.com). However, a comparative test showed that this diagnostic tool’s applicability is limited, and its accuracy is lower compared to other methods, such as the California mastitis test [117,126,127]. Other possible biomarkers for mastitis diagnosis, such as procalcitonin (PCT), neopterin (NPT), haptoglobin (HP), serum amyloid A (SAA), proinflammatory cytokines (IL-1β, IL-8, TNF-α, IF-γ) [128,129,130,131,132,133], as well as lactose [70] are now the subject of research and analysis.

The most widely used method for detecting mastitis, particularly in its subclinical forms, is monitoring the SCC content in milk [101]. When the values of SCC go above the limit, the value of the milk significantly declines. For this reason, researchers consider SCC level to be essential criteria for udder health evaluation [92,134,135,136,137]. Although the direct measurement of SCC level offers great accuracy and reliable information regarding udder health status, this approach may, in some cases, be inaccessible for some dairy farmers and dairy associations due to its high costs (Table 1).

In the past, direct microscopy assessment of the somatic cells was seen as a time-consuming process, whether performed on a single sample or a collection of samples, with uncertain results due to subjective interpretation. Nowadays, due to cutting-edge diagnostic tools such as the DeLaval cell counter, Fossomatic cell counter, PortaCheck^®^, and Somaticell^®^, SCC levels may be evaluated quickly and automatically on many samples [10,138]. Cell counters with high capacity, based on the concept of flow cytometry (fluorooptoelectronic method), such as the Fossomatic cell counter or SomaScope, are often used for measuring SCC in large numbers of samples at once (400–600 samples per hour) [124,139] (Table 1).

### 4.2. Methods Based on the Detection of the Pathogen Agent Causing Mastitis

The diagnostic approaches mentioned in Table 1 provide information regarding the udder’s health status, and some may even indicate the degree to which mastitis has progressed. However, none of them can pinpoint the pathogen agent that is causing the problem. Early and precise detection of the pathogen implicated is associated with a number of benefits, such as appropriate therapy options, including the choice of adequate antibiotics and improved management measures to restrict the spread of disease and antibiotic resistance.

Environmental pathogens that spread predominantly outside the milking parlor account for about 90% of pathogens responsible for udder infections. The most predominant species are *Escherichia coli*, *Streptococcus uberis*, *Streptococcus dysgalactiae*, and *Proteus* spp. [140]. Contagious mastitis is usually caused by pathogens such as *Staphylococcus aureus*, *Streptococcus agalactiae*, and *Mycoplasma* spp., which occur mainly in the cow’s udder, their presence in bulk milk indicating the existence of intramammary infections in the herd [141,142]. Fungi are a less common cause of mastitis, with fewer documented cases, and are most often seen on farms with poor environmental and sanitary conditions [143,144,145,146]. Contamination with microalgae from *Prototheca spp,* frequently related to poor milking conditions and extended antibiotic medication, has also been documented [147,148,149].

The culture-based technique has long been the gold standard for identifying mastitis pathogens. To stimulate growth, a known amount of milk, from either bulk tank or udder quarter, is incubated on culture plates for about 18 h at set temperatures. After the growth phase is over, colony-forming units (CFU) are counted, and the colony phenotype is analyzed to identify the pathogens. Additional biochemical testing may be performed if required. Most pathogens grow well on conventional culture medium, either under aerobe (the vast majority) or under anaerobe conditions (e.g., *Mycoplasma* spp.). The principal disadvantages of bacterial culture are associated with the need for sterile conditions to prevent the development of bovine mastitis non-related microorganisms, the requirement for special equipment, and the need for competent operators to accurately conduct the microbiological procedures and interpret the phenotypic findings. Furthermore, the approach often requires lengthy growth periods (up to 48 h) and is prone to false negatives, with a reported probability of 20–50% [150].

Over the past years, several types of on-farm culture plates were specially designed for farmers and veterinarians, providing a rapid, simple, and low-cost method for determining the probable bacterial etiology of mastitis. While some of the on-farm culturing systems can distinguish only between the main two types of pathogens, Gram-negative and Gram-positive, others may stimulate the development of specific microorganisms and reduce the incubation time using a selective culture medium. For instance, the Accumast^TM^ system separates staphylococci, streptococcus, and Gram-negative bacteria using a tri-plate containing three different chromogenic media. A noticeable color shift is produced when particular bacterial enzymes break chromogens contained in the culture medium [124]. The Minnesota Easy^®^ Culture System uses three different kinds of culture media, Factor™, MacConkey, and Focus™, to differentiate between Gram-positive, and Gram-negative, respectively, *Streptococcus* and *Streptococcus*-like bacteria [151]. Likewise, ClearMilk Test culturing systems enable specialists to identify the pathogen in roughly 22 h using a tri-plate-based culturing system designed to distinguish between *Staphylococcus* spp., *Streptococcus* spp., Gram-negative, as well as yeast [152].

However, although these on-farm culturing systems have become commercially available at reasonable prices, some of the studies have pointed out that the commercial on-farm culturing systems differed significantly in their ability to classify bacterial colonies by genus and species [153] and training beyond the instruction manual is required for untrained observers to make this type of systems effective for pathogen-based mastitis control [154].

Furthermore, given the frequency of false negatives with culture-based methods, the development of molecular diagnostic tests with high test sensitivity and specificity, as well as the necessity to detect non-viable bacteria, has been approached by different researchers that demonstrated effective PCR-based amplification and identification of mastitis pathogens [155,156,157,158,159,160]. Polymerase chain reaction (PCR) is known to be highly sensitive and specific for detecting mastitis pathogens, providing accurate pathogen identification, including those that do not grow using conventional culturing techniques. Although the results when using PCR may be obtained in a matter of hours, a study conducted by Hiitiö et al. (2015) [156] concluded that when low DNA levels have been identified in milk samples, the clinical importance of the data should be carefully reviewed before making any further judgments.

Due to sterility standards, the requirement for sophisticated equipment, and skilled staff, PCR is challenging to deploy on-farm. Furthermore, the presence of recognized PCR inhibitors such as calcium, fat, or protein in milk necessitates using specific DNA extraction techniques to ensure high-quality findings. Alternative to regular PCR and quantitative (qPCR) procedures, loop-mediated isothermal amplification (LAMP) has been described as a promising molecular tool for quick on-farm diagnostics [161,162] and food pathogen detection [163,164,165,166,167]. This approach is quicker than PCR, less costly, highly selective for the target sequence, and requires less template quality and complicated apparatus. Finally, as an isothermal amplification approach, it might be used in the field, needing just a water bath or heat block for the reaction to take place [168,169]. LAMP tests for common mastitis pathogens such as *Staphylococcus aureus*, *Streptococcus agalactiae,* or *Streptococcus uberis* have been developed and validated [170,171,172].

As next-generation sequencing (NGS) is becoming more accessible and less expensive, a new opportunity for developing novel genotyping tools to detect mastitis infectious pathogens arises. Studies have shown that target-specific primers for PCR-mediated amplification with NGS technology to enrich and accurately sequence pathogen genomic areas of interest may contribute to identifying pathogens that were overlooked by other methods [173,174,175]. This outcome indicates the NGS’s practicability and suggests that it is possible to integrate this technique as a diagnostic tool into a veterinary diagnostic laboratory in a cost-effective manner and that in the near future, NGS sequencing can be used as a tool in routine identification of mastitis-related microorganisms [124].

### 4.3. Emergent Methods for Monitoring Udder Health: Infrared Thermography, Biosensors, and Lab-on-Chip Devices

The main barrier to adopting new diagnosis tools is the challenge related to their implementation without disrupting the technological flow in large and medium-sized herds. The even more significant challenge is their incorporation into the technical flow of intensive, free-stall farms. This is why, despite its advantages, the usage of “cow side” tests has decreased in practice as intensive dairy farming has progressed [10,176]. In the context of intensive dairy farming, the traditional method of hand-milking has been mostly phased out in favor of either automated or machine milking. Subsequently, automatic detection techniques for bovine mastitis based on biosensors and employing appropriate sensing technology, such as in-line monitoring of somatic cell count (ISCC) along with quarter-based electrical conductivity (EC) of milk, were developed for the assessment of udder health and early detection of mastitis in large-scale farms [177,178,179]. Precision livestock farming, which makes use of a broad range of technologies, but also incorporates increasingly cutting-edge technologies such as microfluidics, sound analyzers, image-detection, sweat, and salivary sensing, pH and temperature determinations, or serodiagnosis, is becoming one of the most influential and practically applicable in the animal health sector. Biosensors and wearable technologies are now considered state-of-the-art in dairy health management [180].

Biosensors are devices that combine a biological component known as a bioreceptor with a physical transducer known as a sensor. These devices are at the junction of biology and microsystems technology [181]. When a biological recognition element interacts with a target analyte, a quantifiable signal is generated due to the interaction. This signal may then be translated into data by an integrated transducer. There are many different kinds of transducing principles, but the ones that are most frequently researched and used for biomarker and pathogen detection are electrochemical [182,183], optical [184,185], surface plasmon resonance (SPR) [186,187,188], and piezoelectric [189]. Other sensors include acoustic, magnetic, calorimetric, and gravimetric measurement devices [190,191].

Recent developments in microtechnology and nanotechnology have paved the way for improving analytical systems. According to Pérez-López and Merkoci (2011) [192], the foundation of more integrated biosensors for in situ food analysis may be found in improved microfabrication techniques and novel nanomaterials with enhanced sensing capabilities or coupled to biomolecules to work as reporters or signal amplification systems. It has been demonstrated that the incorporation of nanostructures such as carbon materials (for example, nanotubes and graphene sheets), metal nanoparticles (for example, gold, silver, and metal oxides) in various shapes (for example, beads, rods, wires, and discs), and many other structures may promote better signal transduction, assist in biorecognition, and enhance signal amplification.

Identifying the pathogen agent that causes the disease is a paramount step for the successful management of bovine mastitis because it enables veterinarians to lower the risk of developing chronic infections and plan accordingly the antibiotic treatment that will be provided to the animals. For this reason, researchers have orientated their attention to developing fast and user-friendly diagnosis tools for molecular detection, based on either nanotechnology or microfluidics, which may be used “cow-side” and offer an accurate result in a very short amount of time without the milk sample requiring complex processing. For instance, Duarte et al. (2016) [193] designed a magnetic counter that may detect the presence of *Streptococcus agalactiae* (Group B Streptococci) in raw milk. An integrated microfluidic platform was used for the detection process. On this platform, magnetoresistive sensors were employed to dynamically detect magnetic beads of 50 nm in diameter connected to *Streptococcus agalactiae*. Deb et al. (2022) [194] developed an amplification-free visual assay for rapid and sensitive detection of *E coli*. based on numerous gold nanoparticles (AuNPs) trapped on a magnetic microbead surface. The assay was performed without expensive equipment and could detect bacterial DNA as small as 10^2^ CFU/μL [194].

Coatrini-Soares et al. (2022) [195] on the other hand, used machine learning with decision tree models in the development of a low-cost microfluidic-based electronic tongue for the detection of bovine mastitis. The electronic tongue was manufactured using biocompatible molecular architecture and could identify *Staphylococcus aureus* in milk samples with 100% accuracy. Over the past years, different point-of-care (POC) tests were developed for the diagnosis of bovine mastitis [196,197] and further on, research on this topic is currently being carried out in different EU-funded projects [198,199].

Additionally, novel diagnostic methods such as infrared thermography (IRT) have proven to be effective in evaluating udder health and identifying quarters with subclinical mastitis [200]. IRT is an easy-to-use, efficient, cow-side, and noninvasive diagnostic tool that uses infrared imaging and a measurement camera to assess the invisible infrared energy (radiation) emitted by skin or udder surface by converting it to thermal images or thermograms [201]. The very sensitive thermal camera of the IRT can detect even minute shifts in surface temperature or inflammation of the udder. When combined with the mobile-based application, the IRT may transform into a diagnostic tool that is both easy and portable [202]. In their 2018 study, Zaninelli et al. [203] assessed the potential of IRT in the diagnosis of mastitis and found that it correlates very well with the somatic cell count.

This method has been reported to have diagnostic sensitivity and specificity comparable to CMT, distinguishing between clinical and subclinical mastitis in large and small ruminants [204]. Thereby, with further refinements and developments, the IRT has the potential to become a beneficial and practical tool for use on farms in the future [205,206,207] since it is both farmer-friendly and non-invasive. It may enable farmers to assess the milk quality in the first phase of its way (intramammary). Determinations may be made for each mammary compartment separately, with increased local temperature indicating inflammation. Thereby, mixing regular milk with mastitic milk and overall quality deterioration and potential food-borne diseases may be avoided.

### 4.4. Discussions

On a farm, it is essential that the variation in milk SCC from all of the animals that are housed in natural conditions be collected and processed efficiently. Any variation from these changes should be closely analyzed, and the appropriate procedures should be performed to keep the milk quality at its optimal level. SCC may be an effective management instrument for increasing herd immunity, boosting milk production and quality, and enhancing cow health and welfare.

## 5. Conclusions

Climate change substantially impacts the sustainability of food production systems, either domestic or global. Such a challenge requires the adoption of different measurements to sustain agricultural production to fulfill the demand of the rising population. Dairy production will be crucial in feeding the planet’s estimated 9.6 billion population by 2050. As a result, increasing dairy production and animal welfare is an important component in designing future policies that aim to ensure food security, particularly in developing countries.

Many physicochemical and biological tests have been envisioned. They are now routinely utilized worldwide to monitor udder health and prevent the delivery, processing, and marketing of milk and other dairy products that are inadequate in terms of quality. The tests are usually employed following the milk way, from the milking machine to the store shelf. To counteract the detrimental effect of heat stress on mammary gland health and milk quality, a complex, carefully structured program that includes early detection of mastitis, routine monitoring of udder health, and appropriate treatment for mastitis cases must be implemented in dairy farms.

In addition to traditional cow side tests, such as the California Mastitis Test, several other assays provide useful information regarding the variations of certain parameters, specific in the case of mammary inflammations, such as the variations in the somatic cell count, optical density, homogeneity, color, electroconductivity, concentration in certain enzymes or other chemical compounds, as well as the local temperature, may be used to monitor udder health and assess the inflammatory state of the udder.

Ideal diagnosis tools for monitoring udder health should be quick and simple to use and interpret without affecting working flows and daily operations. The diagnostic tools’ specificity, accuracy, and economic accessibility are also paramount. Emergent non-invasive diagnosis tools, such as infrared thermography and biosensor-based devices, may promote animal health and welfare, as well as the quality and safety of dairy products, without hindering the technological flow while providing significant benefits to farmers, manufacturers, and consumers.

## Figures and Tables

**Table 1:** Summary of primary conventional methods, based on physicochemical milk modifications, used for monitoring udder health.

Method	Type (on Farm/ on Laboratory)	Principle	Advantages	Drawbacks
Teat-end scoring	On-farm	Assessment of teat hyperkeratosis using a four-grade chart: Normal—normal appearance, with no ring around the teat canal; Smooth—slightly visible ring with no keratin strands; Rough—a thickened ring that extends between one and three millimeters from the orifice. Scattered fragments of old keratin and disintegrated epithelial cells are visible; Very Rough—a high ring with scattered fragments of old keratin reaching more than 4 mm is visible. The edge of the ring is uneven and shattered, creating a look similar to that of a flower.	Low cost No equipment needed Offers information regarding the effectiveness of the milking machine and milking routine	Requires skilled personnel and attention to scoring
Electric conductivity	On-farm	Changes in the ionic content of milk caused by tissue injury induced by mastitis are measured.	Portable devices allow for cow-side testing Cheap costs Commercially available (Draminski; Milk checker etc.)	The method does not offer information regarding the pathogen that is causing the problem Portable format is less sensitive than other tests based on assessing the number of somatic cells Lower diagnostic sensitivity, especially in bulk tank samples
pH	Lab	The pH in milk samples assessed. Normal milk has a pH of 6.8, while in udder inflammation, the pH tends to become alkaline.	Easy to use Commercially available (pH meters; pH paper with bromothymol blue)	Does not provide information regarding the pathogen that is causing the problem Low sensitivity
California Mastitis test	On-farm	The number of somatic cells (SC) in milk is estimated using an indirect indicator. The test reagent (Bromocresol-purple in the detergent used as a reagent) forms a gel by reacting with the DNA of the cell. The gel viscosity is linked to the amount of SC in the milk sample. The thicker the gel, the larger the number of cells in the milk sample	Methods based on two criteria: number of somatic cells and milk ph. It is possible to perform cow-side Easy to use Low cost All quarters may be tested at the same time Commercially available	Interpretation may be subjective Sensitivity is influenced by the germs that cause the infection.
Detection of enzymatic activity	Lab/ On-farm	Detects variations in color as a means of determining the level of LDH activity.	Fast Easy to use, portable Commercially available	Does not provide information regarding the causative pathogen Poorer performance in terms of diagnosis in comparison to other somatic cell count-based tests
Direct microscopic determination of somatic cells	Laboratory	Identifying epithelial and leucocyte cells discharged into the milk by specific staining	Assessment of the SC contained in milk via direct visual observation	Diagnostic specificity is hampered since a high SC level might be caused by a variety of physiological circumstances. Does not give any information on the pathogen that was responsible for the illness. Requires trained personnel and time consuming Highly subjective
Automatic determination of somatic cells (fluoro optoelectronic method)	Laboratory	The nuclear DNA of somatic cells is stained using a fluorescent dye	Automated Rapid results Commercially available in both portable and high throughput format Increased diagnostic sensitivity and specificity	Requires investment in equipment, which depending on the type, may be highly expensive Trained personnel to operate the equipment Expensive reagents and operating maintenance costs

## Data Availability

Not applicable.
